# Cerebral venous thrombosis: clinical predictors and emerging treatments

**DOI:** 10.1007/s00415-020-10180-3

**Published:** 2020-09-04

**Authors:** A. Al-Ansari, N. P. Robertson

**Affiliations:** 1 Department of Neurology, University Hospital of Wales, Heath Park, Cardiff, CF14 4XN UK; 2 Department of Neurology, Division of Psychological Medicine and Clinical Neuroscience, Cardiff University, University Hospital of Wales, Heath Park, Cardiff, CF14 4XN UK

## Introduction

Cerebral venous thrombosis (CVT) is a distinct form of stroke primarily affecting young- and middle-aged adults and is a commonly considered differential diagnosis in patients presenting with headache and/or neurological deficit particularly in the context of established risk factors. In general, CVT has a relatively favourable long-term prognosis but challenges remain regarding timely diagnosis and appropriate treatment selection, although a consensus of current published guidelines support the use of anticoagulation. However, development of clinical prediction scores, and expanding knowledge of treatment efficacy, may help to further improve clinical management.

This month’s journal club explores three papers relating to CVT. The first paper describes a prospective study of the predictive value of clinical variables and D-dimer levels in patients with clinically possible CVT. The second paper compares endovascular treatment with medical management versus standard anticoagulation for severe CVT. The third paper examines the safety and efficacy of dabigatran etexilate versus warfarin in patients with CVT.

## Prediction of cerebral venous thrombosis with a new clinical score and D-dimer levels

Clinical prediction scores incorporating D-dimer levels are widely used in the evaluation of patients with suspected deep venous thrombosis or pulmonary embolism. This paper describes the development of an equivalent clinical score for stratifying patients into groups with low, moderate and high probability of CVT, and increasing the score’s predictive value by adding D-dimer levels.

Between September 2009 and February 2016, adult patients presenting to the neurological emergency departments of the University Hospitals of Bern and Amsterdam with clinically possible CVT were evaluated by a trained neurologist and consented to participate in the study. Inclusion criteria included one or more of isolated unexpected headache, headache with focal neurological deficits, headache associated with seizure and/or unexplained papilloedema. Exclusion criteria comprised anticoagulation treatment prior to admission and deep venous thrombosis, pulmonary embolus, stroke or myocardial infarction in the 3 months prior to admission. Baseline characteristics, demographic data, risk factors and clinical findings were recorded. Plasma samples for standard parameters and D-dimer levels were taken on admission and analysed by blinded laboratory investigators. Diagnosis of CVT was confirmed by MR and/or CT venography.

359 adults were included in the final analysis, with 94/359 (25.8%) having a confirmed CVT on neuroimaging. All baseline characteristics, demographic data, risk factors and clinical findings were compared between CVT versus non-CVT patients and analysed for statistical significance. Using multivariate logistical regression, six variables were identified as optimal estimates of CVT probability, and these defined the final CVT score with a maximum of 14 points. These included seizure at presentation (4 points), known thrombophilia (4 points), oral contraception (2 points), duration of symptoms greater than 6 days (2 points), worst headache ever (1 point) and focal neurological deficit at presentation (1 point). In the final score, 0–2 points defined those with low CVT probability, 3–5 moderate probability and 6–14 high probability. The score’s predictive value was enhanced by applying D-dimer cut-offs of greater than 500ug/L and 675ug/L, respectively, to the low-, moderate- and high-probability groups. None of the patients with confirmed CVT in the study showed low clinical probability for CVT and D-dimers $<$ 500ug/L.

## Comment

This study presents a new clinical score which may be helpful for prediction of CVT. Strengths include the multicentre design, large sample size, and consideration of a number of risk factors and clinical findings for CVT. Limitations include the development of the score at tertiary centres for cerebrovascular disease and not in standard emergency settings. This may explain the high rate of CVT in the study population. The exclusion of patients with a previous deep vein thrombosis or pulmonary embolus or those already established on anticoagulation may have ruled out a subset of patients with prothrombotic states presenting with a CVT. The score’s utility in standard emergency care departments has yet to be proved.

Heldner et al. (2020) Neurology 10.1212/WNL.0000000000009998

## Effect of endovascular treatment with medical management vs standard care of severe cerebral venous thrombosis

This randomised clinical trial was conducted across eight hospitals in the Netherlands, China and Portugal between September 2011 and December 2017. Adult patients with radiologically confirmed CVT with high probability for poor outcome were included in the study. Risk factors for poor outcome were defined as the presence of at least one of mental status disorders, Glasgow coma scale < 9, intracerebral haemorrhage, or thrombosis of the deep cerebral venous system. Exclusion criteria included thrombocytopenia, space-occupying lesion and clinical and radiological signs of impending trans-tentorial herniation.

Of 67 patients enrolled, 33 (49%) were randomised to receive endovascular treatment with standard medical care (intervention group) and 34 (51%) to receive guideline-based medical care (control group). Patients in the intervention group underwent endovascular treatment within 24 h of randomisation. Intervention consisted of mechanical thrombectomy, pharmacological thrombolysis or both, at the discretion of the interventional radiologist. All patients in the intervention group received therapeutic dose heparin after endovascular treatment was completed. Patients randomised to the control group received therapeutic dose heparin as per international guidelines. All patients in the intervention and control group received long-term anticoagulation with vitamin K antagonists for a variable duration of 3–12 months.

Primary end point was the proportion of patients who had recovered without disability at 12 months. Secondary end points were the proportion of patients with a good recovery at 6 and 12 months, and recanalization rates. Safety end points included symptomatic intracranial haemorrhage. Functional outcome was scored using the modified Rankin Scale by blinded clinicians. At 12 months of follow-up, 22 intervention patients (67%) had achieved the primary end point compared with 23 control patients (68%) (relative risk ratio 0.99; 95% CI, 0.71–1.38). There was no statistically significant difference in mortality or frequency of symptomatic intracerebral haemorrhage between the intervention and control group. The study was prematurely terminated following an interim futility analysis.

## Comment

This is the first randomised clinical trial relating to the efficacy and safety of endovascular therapy on CVT. Strengths of the study include a diverse patient population and inclusion of a number of risk factors for poor outcome. Limitations include the small sample size such that the study was underpowered to detect differences between the intervention and control groups, and the exclusion of the sickest patients with CVT and impending herniation. The authors concede that at the time of the study, available techniques and devices to achieve optimal recanalization in patients may have been sub-optimal, and that future studies using more advanced techniques may identify better recovery rates.

Coutinho et al. (2020) AMA Neurol. Published online May 18, 2020. 10.1001/jamaneurol.2020.1022

## Safety and efficacy of dabigatran etexilate vs dose-adjusted warfarin in patients with cerebral venous thrombosis

The objective of this exploratory randomised trial was to compare safety and efficacy of dabigatran, a direct thrombin inhibitor, with warfarin, a vitamin K antagonist, in the prevention of recurrent thrombo-embolic events in patients who have experienced a CVT. This multicentre trial was conducted between December 2016 and June 2018 at tertiary centres in nine countries in Asia and Europe.

Adult patients with radiologically confirmed CVT were randomised 5–15 days after initial treatment to receive either Dabigatran 150 mg twice daily or Warfarin at a therapeutic dose for 24 weeks. Primary outcome was the number of patients with major haemorrhage or new thromboembolic event during the treatment trial. Secondary outcomes included recanalization rates assessed by MR venography.

120 patients were randomised equally to the 2 treatment groups. Baseline characteristics including demographics, neuroimaging findings, clinical signs and risk factors for CVT were well matched in both groups. 88% of patients in the dabigatran group and 93% in the warfarin group completed treatment over 24 weeks. All 120 patients were included in the final analysis. No recurrent venous thromboembolic events were observed in either group. Three major bleeding events were noted: one (1.7%) case of intestinal bleeding in the dabigatran group and two (3.3%) subdural haemorrhages in the warfarin group. Cerebral recanalization was noted in 33 out of 55 patients (60%) in the dabigatran group (95% CI, 45.9–73.0) and in 35 out of 52 patients (67.3%) in the warfarin group 95% CI, 52.9–79.7).

## Comment

This was an exploratory trial demonstrating low risk of recurrent venous thromboembolism and of clinically relevant bleeding with either dabigatran or dose-adjusted warfarin in the study population. However, due to the small sample population and the low frequency of recurrent venous thromboembolism after CVT, the study was not powered to detect non-inferiority between the two treatment groups.

Ferro et al. JAMA Neurol. 2019; 76(12):1457–1465

## Conclusion

The first paper outlines the development of a scoring system which could be utilised as a pre-test score in the clinical assessment of suspected CVT. The second and third papers demonstrate encouraging work regarding the efficacy and safety of interventional and oral treatments for CVT. Further study on the efficacy of endovascular treatment for CVT using newer techniques and devices is desirable, and may prove of benefit to patients with severe CVT. Larger scale studies are required to demonstrate the equivalence of direct-thrombin inhibitors as an alternative means of anticoagulation in patients with CVT.

